# Ultra-resolution Metagenomics: When Enough Is Not Enough

**DOI:** 10.1128/msystems.00881-21

**Published:** 2021-08-31

**Authors:** Falk Hildebrand

**Affiliations:** a Gut Microbes and Health, Quadram Institute Bioscience, Norwich, United Kingdom; b Digital Biology, Earlham Institute, Norwich, United Kingdom

**Keywords:** metagenomics, sequencing technology, microbiology, microbial evolution

## Abstract

Technological advances in community sequencing have steadily increased the taxonomic resolution at which microbes can be delineated. In high-resolution metagenomics, bacterial strains can now be resolved, enhancing medical microbiology and the description of microbial evolution *in vivo*. In the Hildebrand lab, we are researching novel approaches to further increase the phylogenetic resolution of metagenomics. I propose that ultra-resolution metagenomics will be the next qualitative level of community sequencing, classified by the accurate resolution of ultra-rare genetic events, such as subclonal mutations present in all populations of evolving cells. This will be used to quantify evolutionary processes at ecologically relevant scales, monitor the progress of infections within a patient, and accurately track pathogens in food and infection chains. However, to develop this next metagenomic generation, we first need to understand the currently imposed limits of sequencing technologies, metagenomic strain delineation, and genome reconstructions.

## COMMENTARY

## COMMUNITY SEQUENCING IS A RAPIDLY EVOLVING FIELD

Microbial sequencing has drastically increased our understanding of bacterial communities. Amplicon sequencing (metabarcoding) of the 16S rRNA gene can cost-effectively identify and quantify prokaryotes in communities, but it is limited by sequencing errors that will inflate species diversity if not corrected (1). Further, my group previously described phylogenetic restrictions (2, 3) and taxonomic misclassifications in low-biomass scenarios (4). To optimally account for such errors, we maintain and steadily improve the LotuS2 pipeline (5). However, despite active development in read error correction (6), metabarcoding approaches will remain limited to species-level resolution (7).

**FIG 1:**
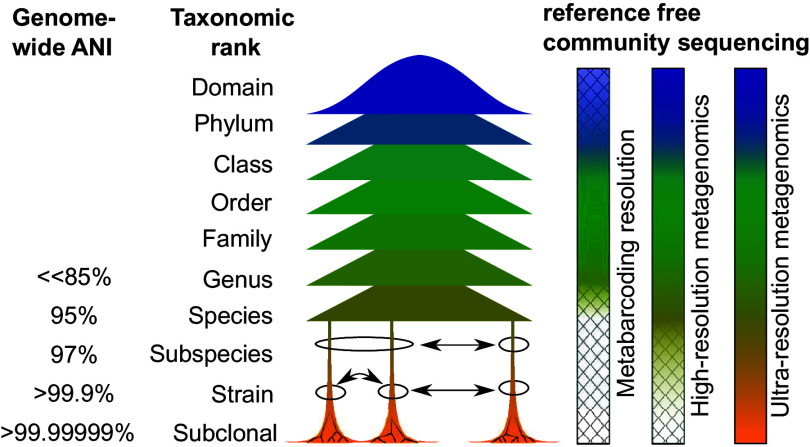
Taxonomic resolution of microbial community sequencing methods. Different taxonomic levels can be approximated by average nucleotide identity (ANI) of its members. Metabarcoding is limited to usually species-level resolution, and different primers bias different kingdoms/phyla. Current high-resolution metagenomics (HRM) can reliably distinguish between species, subspecies, and strains. However, to understand and classify subclonal mutations (single nucleotide variants [SNVs] originating from the same clonal cell within the same microbiome), new approaches are needed to enable ultra-resolution metagenomics (URM). ANI estimates are modified from reference 34; some of these estimates are still the subject of discussion.

There is a strong interest to further resolve microbiomes; currently, bacterial strains can be differentiated using high-resolution metagenomics (HRM) (8, 9). But each population of (clonally derived) cells contains *de novo* mutations segregating in only a fraction of the population—subclonal mutations. These are vital to evolution, as they are either lost or fixed within a population, depending on partly luck and partly relevance to survival. Detecting and linking these to their genome (“phasing”) using reference-free metagenomics will require new approaches, a qualitative leap I refer to as ultra-resolution metagenomics (URM) (Fig. 1). I discuss in this commentary potential use cases of URM and technical challenges to overcome.

## APPLICATIONS OF ULTRA-RESOLUTION METAGENOMICS

Contrary to intuitive assumptions, microbial evolution can happen within days: some gut bacteria adapt to antibiotic treatments within days (10), and plant commensals adapt on an “agriculturally relevant evolutionary timescale” (11). Pseudomonas aeruginosa typically loses dozens of genes during cystic fibrosis infections lasting years (12), Bacteroides fragilis adapts to its specific human host (13), and detecting parallel evolution in pathogens can be used to identify pathogenicity genes (14), as demonstrated using variants of metagenomic and isolate sequencing. These evolutionary processes can be quantified and predicted using population genetics, dependent on accurate genetic information. In cross-sectional or longitudinal intervention studies, we can develop a new holistic eco-evolutionary understanding of microbes under different external circumstances (reviewed in reference 15).

In medical applications, detecting subclonal mutations is vital to predict and avoid treatment resistances, as used in cancer genetics (16). Awareness of these mutations is also relevant in infections; e.g., HIV treatments are combinatorial to avoid subclonal resistance mutations to become prevalent, and a higher frequency of subclonal mutations was linked to prolonged severe acute respiratory syndrome coronavirus 2 (SARS-CoV-2) infections (17). Subclonal mutations further helped disentangle SARS-CoV-2 transmission chains among multiple hosts (17). Using ancient metagenomes, the divergence times of modern gut bacteria were predicted (18). In URM, much more closely related genomes could be used to time transfer events. Thus, a combination of mutation rates and *de novo* and subclonal single nucleotide variants (SNVs) obtained from URM could be used to trace “transfer chains” in hospitals, care homes, or production plants, reconstructing the routes and timing of bacterial colonization. However, currently, this resolution is not available in metagenomics.

## PERFORMANCE OF HIGH-RESOLUTION METAGENOMICS

Short read (e.g., Illumina) sequencing enables HRM through a combination of large read numbers and a relatively good quality. Illumina reads typically have 10^−3^ to 10^−4^ errors/nucleotide, meaning that 1 in 1,000 to 1 in 10,000 nucleotides (nt) are wrong. In large sequencing runs with typically billions of bases, this accumulates to millions of reported errors. Dedicated SNV calling tools can correct some of these but are limited by available computational resources, nonrandom errors in Illumina reads, and mapping biases (19). Thus, sequencing error rate does not directly translate to metagenomic resolution.

The earliest HRM approaches were reference based; i.e., sequencing reads were mapped to reference genomes to identify SNVs per species and metagenome (20). However, reference-based metagenomics has several disadvantages, primarily that the majority of unknown microorganisms is not represented in reference databases (21, 22). Further, mapping to a distantly related reference genome will bias and increase the SNV calling error (23). Last, the bacterial genome is very dynamic: our experiments showed that on average, only 72% of genes are shared among intraspecific strains from 155 bacterial species (24). This might also apply to gut bacteria: when we *de novo* reconstructed the genome of Parabacteroides distasonis, this genome was better covered in time series metagenomes from the same host (median, 98%) than those from other hosts (<80% coverage).

For the same species, two extremely similar strains were detected, occurring in cohousing family members. These strains were only 185 nt distant (99.996% average nucleotide identity [ANI]) but could be reliably assigned to dozens of time series samples from their respective human hosts. Thus, using *de novo*-reconstructed genomes, we could effectively resolve strains at <4 × 10^−4^ errors/nucleotide (25). This workflow is implemented in our MATAFILER pipeline and was recently used to track 440 species in 5,278 metagenomes (9). To estimate the metagenomic resolution on real data, we compared the nucleotide sequence of >26,000 strains persisting between longitudinal samples. The average ANI was 99.98% (estimated error rate, 2 × 10^−3^), although 55% of these strains were reconstructed at 100% ANI (9). This resolution compares favorably to other strain-level pipelines when evaluated on real metagenomes (see Fig. 2d in reference 26), exemplifying resolution of current HRM.

HRM typically use a “consensus” SNV calling approach, considering only the major (>0.5 frequency) alleles in a population. Typically, samples are excluded if ≥2 conspecific strains of a species are present. In contrast, HRM approaches such as STRONG phase SNVs to reconstruct strain-specific genomes, even from mixtures of conspecific strains co-occurring in the same samples, delineating strains at 99.95% ANI (5 × 10^−3^ errors/nucleotide) (27). In a different strategy, inStrain (26) reports minor and major alleles indiscriminately, reporting high strain similarities in simulations (99.9998% ANI, 2 × 10^−5^ errors/nucleotide) but also reporting sequencing errors.

To distinguish true biological variation from sequencing errors is one of the biggest challenges to enabling URM.

## ENABLING ULTRA-RESOLUTION METAGENOMICS

The bacterial mutation rate is estimated at between 10^−8^ and 10^−5^ mutations/nucleotide/year (28), and population genetics theory predicts that neutral mutations get fixed at a similar rate. To identify bacterial *de novo* mutations fixed after 1 year would thus require a metagenomic resolution of <<1e−5 errors/nucleotide. Detecting subclonal mutations at a minor allele frequency (MAF) of ≤0.5 would require a similar error rate but a much higher read coverage, approximated recently at >5× coverage for Illumina reads (26). Therefore, URM requires a combination of (i) increased sequencing coverage, (ii) read accuracy, (iii) phasing of SNVs, and (iv) using orthogonal genetic markers, as I briefly discuss below.

Increased read coverage is enabled through inexpensive, high-throughput sequencing. However, to cover more species from an ecosystem, a linear increase in sequencing depth is unlikely to cover its rare members, as species abundance curves in ecosystems follow a power law. We are therefore exploring alternatives to systematically increase the coverage (and thereby resolution) of underrepresented microbes. One of our bioinformatic solutions is to fine-tune strain delineation at ≥2× coverage (9). Experimentally, we demonstrated the potential of microfluidics to enrich particular microbes (29), while single-cell sequencing can increase read coverage of a single cell, allowing phasing of subclonal mutations.

Simply increasing sequencing coverage does not scale linearly to SNV calling accuracy, since PCR and Illumina sequencing errors are not random (30). Single-molecule sequencing like PacBio has the advantage of having random read errors, allowing for better error correction. This is implemented in PacBio HiFi sequencing, reaching under optimal conditions an error rate below 10^−5^. Illumina read accuracy can be further increased to potential error rates of ≤10^−7^ using molecular barcoding techniques (31). Bioinformatically, read error profiles can be generated to further increase accuracy, as used in DADA2 (6). In metagenomes this is not applicable, but instead, spiking artificial DNA into a sample can be used to calculate sample-specific error profiles.

Phasing SNVs is possible with short read sequencing but inefficient when very similar genomes are co-occurring. In the URM context, other techniques need to be employed, such as single-cell sequencing or barcoding DNA molecules (32). However, using long read sequencing (Oxford Nanopore or PacBio) at high read accuracy might be the most straightforward approach.

Instead of SNVs, structural variants (SVs) of microbial genomes can be used to track bacterial evolution. Even short read data can reliably track small SVs such as indels and microsatellites, and the confidence in observing a specific, e.g., 5-bp, deletion across samples is significantly higher than repeatedly observing a single SNV. SVs can inform on the evolution of bacteria; for example, we used microsatellites to reconstruct the demography and spread of Mycobacterium tuberculosis (33). With long read technologies, the detection of larger inversions, insertions, and deletions will become more commonplace, allowing increased resolution of evolutionary processes.

## CONCLUSION

Ultra-resolution metagenomics (URM), here defined by the ability to detect ultra-rare mutations within a microbial population, is the next logical step in metagenomics. For evolutionary and epidemiological science, this resolution will revolutionize our understanding of microbial ecosystems. Given the importance of this technology, the work of my group is focused on providing both experimental and bioinformatic solutions to get the most out of current HRM and future URM data. Given the rapidly increasing accuracy and throughput of long read sequencing, I predict that standard metagenomic sequencing data will be URM compatible within 5 years.

## References

[1] Quince C, Lanzén A, Curtis TP, Davenport RJ, Hall N, Head IM, Read LFF, Sloan WT. 2009. Accurate determination of microbial diversity from 454 pyrosequencing data. Nat Methods 6:639–641. doi:10.1038/nmeth.1361. 19668203

[2] Tedersoo L, Anslan S, Bahram M, Põlme S, Riit T, Liiv I, Kõljalg U, Kisand V, Nilsson H, Hildebrand F, Bork P, Abarenkov K. 2015. Shotgun metagenomes and multiple primer pair-barcode combinations of amplicons reveal biases in metabarcoding analyses of fungi. MycoKeys 10:1–43. doi:10.3897/mycokeys.10.4852.

[3] Bahram M, Anslan S, Hildebrand F, Bork P, Tedersoo L. 2018. Newly designed 16S rRNA metabarcoding primers amplify diverse and novel archaeal taxa from the environment. Environ Microbiol Rep 11:487–494. doi:10.1111/1758-2229.12684. 30058291 PMC6618113

[4] Bedarf JR, Beraza N, Khazneh H, Özkurt E, Baker D, Borger V, Wüllner U, Hildebrand F. 2021. Much ado about nothing? Off-target amplification can lead to false-positive bacterial brain microbiome detection in healthy and Parkinson’s disease individuals. Microbiome 9:75. doi:10.1186/s40168-021-01012-1. 33771222 PMC8004470

[5] Hildebrand F, Tadeo R, Voigt A, Bork P, Raes J. 2014. LotuS: an efficient and user-friendly OTU processing pipeline. Microbiome 2:30. doi:10.1186/2049-2618-2-30. 27367037 PMC4179863

[6] Callahan BJ, McMurdie PJ, Rosen MJ, Han AW, Johnson AJA, Holmes SP. 2016. DADA2: high-resolution sample inference from Illumina amplicon data. Nat Methods 13:581–583. doi:10.1038/nmeth.3869. 27214047 PMC4927377

[7] de Oliveira Martins L, Page AJ, Mather AE, Charles IG. 2019. Taxonomic resolution of the ribosomal RNA operon in bacteria: implications for its use with long-read sequencing. NAR Genom Bioinform 2:lqz016. doi:10.1093/nargab/lqz016. 33575567 PMC7671355

[8] Frioux C, Singh D, Korcsmaros T, Hildebrand F. 2020. From bag-of-genes to bag-of-genomes: metabolic modelling of communities in the era of metagenome-assembled genomes. Comput Struct Biotechnol J 18:1722–1734. doi:10.1016/j.csbj.2020.06.028. 32670511 PMC7347713

[9] Hildebrand F, Gossmann TI, Frioux C, Özkurt E, Myers PN, Ferretti P, Kuhn M, Bahram M, Nielsen HB, Bork P. 2021. Dispersal strategies shape persistence and evolution of human gut bacteria. Cell Host Microbe 29:1167–1176.e9. doi:10.1016/j.chom.2021.05.008. 34111423 PMC8288446

[10] Roodgar M, Good B, Garud N, Martis S, Avula M, Zhou W, Lancaster S, Lee H, Babveyh A, Nesamoney S, Pollard K, Snyder M. 2019. Longitudinal linked read sequencing reveals ecological and evolutionary responses of a human gut microbiome during antibiotic treatment. bioRxiv doi:10.1101/2019.12.21.886093. PMC8327913 34301627

[11] Li E, de Jonge R, Liu C, Jiang H, Friman V-P, Pieterse CMJ, Bakker PAHM, Jousset A. 2021. Rapid evolution of bacterial mutualism in the plant rhizosphere. Nat Commun 12:3829. doi:10.1038/s41467-021-24005-y. 34158504 PMC8219802

[12] Dingemans J, Ye L, Hildebrand F, Tontodonati F, Craggs M, Bilocq F, De Vos D, Crabbé A, Van Houdt R, Malfroot A, Cornelis P. 2014. The deletion of TonB-dependent receptor genes is part of the genome reduction process that occurs during adaptation of Pseudomonas aeruginosa to the cystic fibrosis lung. Pathog Dis 71:26–38. doi:10.1111/2049-632X.12170. 24659602

[13] Zhao S, Lieberman TD, Poyet M, Kauffman KM, Gibbons SM, Groussin M, Xavier RJ, Alm EJ. 2019. Adaptive evolution within gut microbiomes of healthy people. Cell Host Microbe 25:656–667.e8. doi:10.1016/j.chom.2019.03.007. 31028005 PMC6749991

[14] Lieberman TD, Michel J-B, Aingaran M, Potter-Bynoe G, Roux D, Davis MR, Skurnik D, Leiby N, LiPuma JJ, Goldberg JB, McAdam AJ, Priebe GP, Kishony R. 2011. Parallel bacterial evolution within multiple patients identifies candidate pathogenicity genes. Nat Genet 43:1275–1280. doi:10.1038/ng.997. 22081229 PMC3245322

[15] Garud NR, Pollard KS. 2020. Population genetics in the human microbiome. Trends Genet 36:53–67. doi:10.1016/j.tig.2019.10.010. 31780057

[16] Schmitt MW, Kennedy SR, Salk JJ, Fox EJ, Hiatt JB, Loeb LA. 2012. Detection of ultra-rare mutations by next-generation sequencing. Proc Natl Acad Sci USA 109:14508–14513. doi:10.1073/pnas.1208715109. 22853953 PMC3437896

[17] Lythgoe KA, Hall M, Ferretti L, de Cesare M, MacIntyre-Cockett G, Trebes A, Andersson M, Otecko N, Wise EL, Moore N, Lynch J, Kidd S, Cortes N, Mori M, Williams R, Vernet G, Justice A, Green A, Nicholls SM, Ansari MA, Abeler-Dörner L, Moore CE, Peto TEA, Eyre DW, Shaw R, Simmonds P, Buck D, Todd JA, Connor TR, Ashraf S, da Silva Filipe A, Shepherd J, Thomson EC, Bonsall D, Fraser C, Golubchik T, on behalf of the Oxford Virus Sequencing Analysis Group (OVSG). 2021. SARS-CoV-2 within-host diversity and transmission. Science 372:eabg0821. doi:10.1126/science.abg0821. 33688063 PMC8128293

[18] Tett A, Huang KD, Asnicar F, Fehlner-Peach H, Pasolli E, Karcher N, Armanini F, Manghi P, Bonham K, Zolfo M, De Filippis F, Magnabosco C, Bonneau R, Lusingu J, Amuasi J, Reinhard K, Rattei T, Boulund F, Engstrand L, Zink A, Collado MC, Littman DR, Eibach D, Ercolini D, Rota-Stabelli O, Huttenhower C, Maixner F, Segata N. 2019. The Prevotella copri complex comprises four distinct clades underrepresented in Westernized populations. Cell Host Microbe 26:666–679.e7. doi:10.1016/j.chom.2019.08.018. 31607556 PMC6854460

[19] Li H. 2011. A statistical framework for SNP calling, mutation discovery, association mapping and population genetical parameter estimation from sequencing data. Bioinformatics 27:2987–2993. doi:10.1093/bioinformatics/btr509. 21903627 PMC3198575

[20] Schloissnig S, Arumugam M, Sunagawa S, Mitreva M, Tap J, Zhu A, Waller A, Mende DR, Kultima JR, Martin J, Kota K, Sunyaev SR, Weinstock GM, Bork P. 2013. Genomic variation landscape of the human gut microbiome. Nature 493:45–50. doi:10.1038/nature11711. 23222524 PMC3536929

[21] Nielsen HB, Almeida M, Juncker AS, Rasmussen S, Li J, Sunagawa S, Plichta DR, Gautier L, Pedersen AG, Le Chatelier E, Pelletier E, Bonde I, Nielsen T, Manichanh C, Arumugam M, Batto J-M, Quintanilha dos Santos MB, Blom N, Borruel N, Burgdorf KS, Boumezbeur F, Casellas F, Doré J, Dworzynski P, Guarner F, Hansen T, Hildebrand F, Kaas RS, Kennedy S, Kristiansen K, Kultima JR, Léonard P, Levenez F, Lund O, Moumen B, Le Paslier D, Pons N, Pedersen O, Prifti E, Qin J, Raes J, Sørensen S, Tap J, Tims S, Ussery DW, Yamada T, Renault P, Sicheritz-Ponten T, Bork P, Wang J, Brunak S, Ehrlich SD, MetaHIT Consortium. 2014. Identification and assembly of genomes and genetic elements in complex metagenomic samples without using reference genomes. Nat Biotechnol 32:822–828. doi:10.1038/nbt.2939. 24997787

[22] Gilroy R, Ravi A, Getino M, Pursley I, Horton DL, Alikhan N, Baker D, Gharbi K, Hall N, Watson M, Adriaenssens EM, Foster-Nyarko E, Jarju S, Secka A, Antonio M, Oren A, Chaudhuri RR, La Ragione R, Hildebrand F, Pallen MJ. 2021. Extensive microbial diversity within the chicken gut microbiome revealed by metagenomics and culture. PeerJ 9:e10941. doi:10.7717/peerj.10941. 33868800 PMC8035907

[23] Bush SJ, Foster D, Eyre DW, Clark EL, De Maio N, Shaw LP, Stoesser N, Peto TEA, Crook DW, Walker AS. 2020. Genomic diversity affects the accuracy of bacterial single-nucleotide polymorphism–calling pipelines. Gigascience 9:653774. doi:10.1093/gigascience/giaa007. PMC7002876 32025702

[24] Maistrenko OM, Mende DR, Luetge M, Hildebrand F, Schmidt TSB, Li SS, Rodrigues JFM, von Mering C, Pedro Coelho L, Huerta-Cepas J, Sunagawa S, Bork P. 2020. Disentangling the impact of environmental and phylogenetic constraints on prokaryotic within-species diversity. ISME J 14:1247–1259. doi:10.1038/s41396-020-0600-z. 32047279 PMC7174425

[25] Hildebrand F, Moitinho-Silva L, Blasche S, Jahn MT, Gossmann TI, Huerta-Cepas J, Hercog R, Luetge M, Bahram M, Pryszlak A, Alves RJ, Waszak SM, Zhu A, Ye L, Costea PI, Aalvink S, Belzer C, Forslund SK, Sunagawa S, Hentschel U, Merten C, Patil KR, Benes V, Bork P. 2019. Antibiotics-induced monodominance of a novel gut bacterial order. Gut 68:1781–1790. doi:10.1136/gutjnl-2018-317715. 30658995 PMC6839795

[26] Olm MR, Crits-Christoph A, Bouma-Gregson K, Firek BA, Morowitz MJ, Banfield JF. 2021. inStrain profiles population microdiversity from metagenomic data and sensitively detects shared microbial strains. Nat Biotechnol 39:727–736. doi:10.1038/s41587-020-00797-0. 33462508 PMC9223867

[27] Quince C, Nurk S, Raguideau S, James R, Soyer OS, Kimberly SJ, Limasset A, Murat Eren A, Chikhi R, Darling AE. 2020. Metagenomics strain resolution on assembly graphs. bioRxiv 10.1101/2020.09.06.284828. PMC8311964 34311761

[28] Duchêne S, Holt KE, Weill F-X, Le Hello S, Hawkey J, Edwards DJ, Fourment M, Holmes EC. 2016. Genome-scale rates of evolutionary change in bacteria. Microb Genom 2:e000094. doi:10.1099/mgen.0.000094. 28348834 PMC5320706

[29] Pryszlak A, Wenzel T, Seitz KW, Hildebrand F, Kartal E, Cosenza MR, Benes V, Bork P, Merten C. 2021. Enrichment of gut microbiome strains for cultivation-free genome sequencing using droplet microfluidics. arXiv 2106.01455 [q-bio.GN]. 10.1016/j.crmeth.2021.100137 PMC8787643 35118437

[30] Li H. 2014. Toward better understanding of artifacts in variant calling from high-coverage samples. Bioinformatics 30:2843–2851. doi:10.1093/bioinformatics/btu356. 24974202 PMC4271055

[31] Salk JJ, Schmitt MW, Loeb LA. 2018. Enhancing the accuracy of next-generation sequencing for detecting rare and subclonal mutations. Nat Rev Genet 19:269–285. doi:10.1038/nrg.2017.117. 29576615 PMC6485430

[32] Tracanna V, Ossowicki A, Petrus MLC, Overduin S, Terlouw BR, Lund G, Robinson SL, Warris S, Schijlen EGWM, van Wezel GP, Raaijmakers JM, Garbeva P, Medema MH. 2021. Dissecting disease-suppressive rhizosphere microbiomes by functional amplicon sequencing and 10× metagenomics. mSystems 6:e01116-20. doi:10.1128/mSystems.01116-20. 34100635 PMC8269251

[33] Wirth T, Hildebrand F, Allix-Béguec C, Wölbeling F, Kubica T, Kremer K, van Soolingen D, Rüsch-Gerdes S, Locht C, Brisse S, Meyer A, Supply P, Niemann S. 2008. Origin, spread and demography of the Mycobacterium tuberculosis complex. PLoS Pathog 4:e1000160. doi:10.1371/journal.ppat.1000160. 18802459 PMC2528947

[34] Van Rossum T, Ferretti P, Maistrenko OM, Bork P. 2020. Diversity within species: interpreting strains in microbiomes. Nat Rev Microbiol 18:491–506. doi:10.1038/s41579-020-0368-1. 32499497 PMC7610499

